# miR‐218 affects the ECM composition and cell biomechanical properties of glioblastoma cells

**DOI:** 10.1111/jcmm.17428

**Published:** 2022-06-15

**Authors:** Małgorzata Grabowska, Konrad Kuczyński, Monika Piwecka, Alicja Rabiasz, Joanna Zemła, Paweł Głodowicz, Dariusz Wawrzyniak, Małgorzata Lekka, Katarzyna Rolle

**Affiliations:** ^1^ Institute of Bioorganic Chemistry Polish Academy of Sciences Poznań Poland; ^2^ NanoBioMedical Centre Adam Mickiewicz University Poznań Poland; ^3^ Institute of Human Genetics Polish Academy of Sciences Poznań Poland; ^4^ Institute of Nuclear Physics Polish Academy of Sciences Kraków Poland

**Keywords:** AFM, ECM, GBM, glioblastoma, miR‐218, tenascin‐C

## Abstract

Glioblastoma (GBM) is the most common malignant brain tumour. GBM cells have the ability to infiltrate into the surrounding brain tissue, which results in a significant decrease in the patient’s survival rate. Infiltration is a consequence of the low adhesion and high migration of the tumour cells, two features being associated with the highly remodelled extracellular matrix (ECM). In this study, we report that ECM composition is partially regulated at the post‐transcriptional level by miRNA. Particularly, we show that miR‐218, a well‐known miRNA suppressor, is involved in the direct regulation of ECM components, tenascin‐C (TN‐C) and syndecan‐2 (SDC‐2). We demonstrated that the overexpression of miR‐218 reduces the mRNA and protein expression levels of TN‐C and SDC‐2, and subsequently influences biomechanical properties of GBM cells. Atomic force microscopy (AFM) and real‐time migration analysis revealed that miR‐218 overexpression impairs the migration potential and enhances the adhesive properties of cells. AFM analysis followed by F‐actin staining demonstrated that the expression level of miR‐218 has an impact on cell stiffness and cytoskeletal reorganization. Global gene expression analysis showed deregulation of a number of genes involved in tumour cell motility and adhesion or ECM remodelling upon miR‐218 treatment, suggesting further indirect interactions between the cells and ECM. The results demonstrated a direct impact of miR‐218 reduction in GBM tumours on the qualitative ECM content, leading to changes in the rigidity of the ECM and GBM cells being conducive to increased invasiveness of GBM.

## 1 INTRODUCTION

Glioblastoma (GBM) is the most malignant astrocytic brain tumour. Despite treatment with advanced therapies, including aggressive surgical intervention, radiotherapy, and systemic chemotherapy, as well as significant advances in the field of oncology, the average survival time for GBM patients is approximately 15 months, with a 5‐year survival rate of only 5%.^1^, ^2^ The main factor contributing to this poor prognosis is the ability of GBM cells to infiltrate adjacent tissues, resulting in a high rate of tumour recurrence.^3^ These notable migration and invasion abilities could be explained by alterations occurring in the structure of cancer cells and their surroundings, defined by mechanobiology.^4^

To promote the invasiveness, cancer cells modify not only themselves but also their environment, namely the extracellular matrix (ECM). It consists of over 300 different proteins, including proteoglycans and glycoproteins.^5^ Neoplastic tissues are characterized by the phenomenon of desmoplasia, manifested by the intense formation of a dense ECM consisting of increased levels of total fibrillar collagen, fibronectin, proteoglycans and tenascin‐C (TN‐C).^6^ The capability to synthesize specific and cancer‐related ECM components has been shown to be relevant for the high invasiveness of tumour cells. The changed protein profile within ECM increases the stiffness of cancerous tissue,^7^, ^8^ which may lead to enhanced cell–ECM adhesion through the involvement of local adhesion proteins. The general trend observed for many types of cells indicates that cell spread and adhesion are improved on harder matrices.^9^, ^10^ The effect of the environment on the cells is explained by the mechanotransduction mechanism, in which mechanical and cell‐specific signals are actively detected by cells and converted into intracellular biochemical signals. In this manner, the ECM can affect cancer cell behaviour, including invasion and metastasis.^11^, ^12^ Therefore, cancer should be considered as a disease with alterations in both cells and their microenvironment, including also the biochemical and biophysical properties of the ECM. Not only proteins suspended in the ECM have an impact on the invasiveness of the tumour, but also transmembrane proteins. The syndecans are a four‐member family of evolutionarily conserved small type I transmembrane proteoglycans implicated in the formation of specialized membrane domains, cell adhesion, cytoskeletal organization, migration and wound healing. They have been also related to the pathological conditions, including inflammation and cancer.^13^, ^14^, ^15^ For instance, elevated expression of syndecan‐2 (SDC‐2) has been correlated with increased invasiveness in various types of cancers, including fibrosarcoma,^16^ melanoma,^17^ colon,^18^ pancreatic^19^ and colorectal^20^ cancers, while TN‐C is overexpressed in brain tumours,^21^ breast,^22^ lung^23^ and colorectal^24^ cancers.

In the recent 20 years, microRNAs (miRNAs) have emerged as key regulators of gene expression at the post‐transcriptional level. miRNAs are a large family of endogenous, evolutionarily conserved, non‐coding RNAs that are ~22 nucleotides long, and they have been implicated in the regulation of nearly every biological process.^25^ Deregulated miRNA expression has been shown to play a role in the pathogenesis of a growing list of human diseases, including cancer and cardiovascular, neurodegenerative and autoimmune disorders.^26^, ^27^, ^28^, ^29^ For example, in GBM, it has been already demonstrated that the downregulation of miR‐218 affects cell proliferation, epithelial‐to‐mesenchymal transition,^30^ metabolism of cancer cells^31^ and cancer stem cell properties.^32^ How miRNAs are involved in the regulation of ECM composition and the mechanobiology of cancer cells in GBM tumours is largely unknown. In principle, miRNAs can exert their control over the ECM either directly by targeting mRNAs encoding ECM proteins or indirectly by modulating the expression of genes involved in the synthesis or degradation of ECM molecules. Here, we have evidenced that miR‐218, one of the highly downregulated miRNAs in GBM cells, is involved in the direct regulation of TN‐C and SDC‐2, two highly overrepresented proteins in GBM and ECM components. Both SDC‐2 and TN‐C have been previously demonstrated to increase tumour cell migration and invasiveness. In the course of the study, we attempted to validate how miR‐218 interaction with its ECM targets affects globally a microenvironment and biomechanical properties of GBM cells; we introduced miR‐218 mimic into GBM cells and measured the consequences on the migration, adhesion and stiffness properties of individual cancer cells. As demonstrated by real‐time migration analysis and single‐cell force spectroscopy (SCFS) measurements using contact‐mode atomic force microscopy (AFM), overexpression of miR‐218 had a pronounced effect on the mechanical properties of GBM cells, influencing their migration potential, adhesion and overall stiffness. Collectively, our results indicate that miR‐218 is a potent tumour suppressor in glioma with a substantial impact on the ECM composition and biomechanical properties of GBM.

## 2 MATERIALS AND METHODS

### 2.1 Patient sample collection

The GBM samples (*n* = 19) were obtained from the Clinic of Neurosurgery and Neurotraumatology, Karol Marcinkowski University of Medical Sciences in Poznan, Poland, during 2016–2017 based on the approval from the Ethical Committee (Nr. 46/13), and individuals signed an informed consent form.

### 2.2 Cell culture

Human glioblastoma cell lines U‐118 MG, U‐138 MG, U‐251 MG and T98‐G purchased from American Type Culture Collection (ATCC) were used in the study. Cells were maintained in recommended medium, Eagle’s Minimal Essential Medium (EMEM, Corning) or Dulbecco’s modified Eagle’s medium (DMEM, ATCC) supplemented with 10% foetal bovine serum (FBS, Sigma‐Aldrich) and 1% penicillin–streptomycin antibiotic (Sigma‐Aldrich) and incubated at 37°C and 5% CO_2_ in a humidified atmosphere in an incubator.

### 2.3 Transfection

The cells were transfected with *mir*Vana™ hsa‐miR‐218‐5p mimic (Invitrogen) in a final concentration of 10 nM and 50 nM at 70%–80% confluency. Lipofectamine™ 2000 (Invitrogen) was used as a transfection agent according to the manufacturer’s protocols. A non‐specific scrambled siRNA (Sigma‐Aldrich) was used as a control in all transfection experiments. The cells were processed after 24 h for the quantification of transcript levels using qPCR, Western blot, cellular assays or AFM analysis.

### 2.4 Luciferase reporter assay

The TargetScan (www.targetscan.org) analysis predicted the 3′UTR segments of TN‐C and SDC‐2 interacting with hsa‐miR‐218‐5p. Based on them, 22‐nucleotides‐long fragments were designed, along with corresponding mutants, characterized by one point mutation and one codon change. As a control was used a perfect match sequence, fully complementary to the miR‐218. Oligonucleotides were synthesized by Sigma‐Aldrich. Fragments were then ligated with the pmirGLO Dual‐Luciferase miRNA Target Expression Vector (Promega), transformed by heat shock into TOP10 *Escherichia coli* cells and multiplied. Verified by sequencing, plasmids were transfected together with *mir*Vana™ hsa‐miR‐218‐5p mimic to the U‐118 glioblastoma cell line. Luciferase activity was analysed with Dual‐Glo® Luciferase Assay System (Promega) by the manufacturer’s instructions using the Synergy™ HTX Multi‐Mode Microplate Reader (BioTek).

### 2.5 Western blots

U‐118 MG cells were lysed by sonication for protein isolation in 10 nM Tris–HCl, pH = 7.5 with protease inhibitor cocktail (Sigma‐Aldrich). Protein expression glyceraldehyde 3‐phosphate dehydrogenase (GAPDH) level was used as an endogenous control. For TN‐C, SDC‐2 and GAPDH detection, 25 μg of isolated material was used. Protein was denatured, separated by SDS‐PAGE (SDS‐polyacrylamide gel electrophoresis) on 7,5% for TN‐C and 15% gels for SDC‐2 and GAPDH detection, with electric current 30 mA and wet transferred to the polyvinylidene fluoride membrane using electric current 130 mA, and blocked with 5% skimmed milk. After incubation with primary and secondary antibodies, proteins of interest were detected with Western Bright Sirius Chemiluminescent Detection Kit (Advansta). The following antibodies were used: polyclonal TN‐C H‐300 (dilution 1:500; Santa Cruz Biotechnology), monoclonal SDC‐267088‐1‐Ig (dilution 1:500; Proteintech), monoclonal GAPDH 0411 (dilution 1:500; Santa Cruz Biotechnology) and anti‐mouse A9044/rabbit A6154 peroxidase (dilution 1:10,000; Sigma‐Aldrich). TN‐C and GAPDH antibodies were diluted in 3% bovine serum albumin (BSA, Sigma‐Aldrich), and others, in skimmed milk. The intensity of individual bands was analysed quantitively by Multi Gauge ver. 2.0 (Fujifilm). The relative ratio of protein‐level expression was determined based on the densitometric measurements of band intensities in relation to the control sample.

### 2.6 qRT‐PCR

Total RNA was isolated using the TRIzol reagent (Invitrogen) according to the manufacturer’s protocol. Afterwards, RNA was purified with the DNA‐free™ DNA Removal Kit (Ambion). The reverse transcription reaction was carried out with the Transcriptor High Fidelity cDNA Synthesis Kit (Roche) according to the manufacturer’s protocol, using in each case 500 ng of RNA material. The reverse transcription for miRNA was performed by two‐step miRNA 1st‐Strand cDNA Synthesis Kit (Agilent Technologies). cDNA was used in real‐time quantitative reverse transcription PCR (qRT‐PCR), with the use of LightCycler®480 (Roche), in three technical replicates. Primers with corresponding probes were designed in the Universal Probe Library Assay Design Center (https://qpcr.probefinder.com/organism.jsp). Relative expression was analysed in the LightCycler®480 Software release 1.5.1.62 (Roche). The level of hypoxanthine phosphoribosyltransferase (HPRT) was used as an endogenous control for analysis of extracellular matrix proteins. In case of miR‐218, the level of 18S ribosomal RNA was used for normalization. Sequences of primers 5′‐3′ and list of probes were as follows: TN‐C forward: GGGATTAATGTCGGAAATGGT; TN‐C reverse: CCGGACCAAAACCATCAGT; TN‐C probe: 76; SDC‐2 forward: TTATCAGATGTCAGCTCTGCTCTC; SDC‐2 reverse: GTGGATCCTGCTCACCTTG; SDC‐2 probe: 49; HPRT forward: CGAGCAAGACGTTCAGTCCT; HPRT reverse: TGACCTTGATTTATTTTGCATACC; HPRT probe: 73; miR‐218: TTGTGCTTGATCTAACCATGT; R18 forward: CATTCTTGGCAAATGCTTTCG; and R18 reverse: CGCCGCTAGAGGTGAAATTC. As a control, RNA from normal, healthy brains (Ambion, First Choice® Human Brain Reference RNA, Cat # 6050, whole brain pooled from 10 females and 13 men, Caucasian, age: 23–86) was used.

### 2.7 PCR array of human cell motility, extracellular matrix and adhesion molecules

U‐118 MG cells treated with miR218 mimic were collected for total RNA isolation with ExtractME Total RNA Kit (Blirt) according to the manufacturer’s protocol. 900 ng of RNA was used in the reverse transcription procedure with RT^2^ Easy First Strand Kit (Qiagen). cDNA mixed with the RT^2^ SYBR Green was then evenly aliquoted onto the RT^2^ profiler plates: Human Cell Motility, and Human Extracellular Matrix and Adhesion Molecules (Qiagen). qRT‐PCRs were conducted in LightCycler®480 (Roche), and subsequently analysed by software provided online by Qiagen.

### 2.8 Real‐time migration

Real‐time cell migration monitoring was performed in the xCELLigence® system using the RTCA DP apparatus (ACEA Biosciences). The experiment was carried out on 16‐well CIM‐Plates, in which culture medium enriched with FBS, served as a chemoattractant, was applied into lower part of the CIM‐Plate. To the upper chamber was applied an unsupplemented medium. The first stage of the experiment served to measure the background of electrical impedance. Then, 10,000 U‐118 MG cells treated with miR‐218 mimic were seeded on the upper chamber of the plate. The CIM‐Plate was installed in the RTCA apparatus; from that moment, for further 48 h, the system registered the level of electrical impedance every 15 min. The results of the experiment were presented in the cell index unit of the xCELLigence® system, which corresponded to the measured impedance minus the impedance of the background. The experimental curves were adjusted to the sigmoidal equation and the half‐time effcective migration values (effective time 50, ET 50) were calculated.

### 2.9 Wound healing assay

U‐118 MG cells were grown to achieve 90% of confluency on 12‐well plates and then transfected with miR‐218 mimic. After the medium is changed, scratches were created by scraping cells in a straight line using a 200‐μl tip. From that moment on, for 72 h at 12‐h intervals, pictures of the culture were taken by a Leica DMI4000 B inverted microscope with 5x magnification objective. The analysis of the degree of the individual scratch area was carried out by the Tscratch software version 1.0 (CSElab). That software is based on novel algorithm for measuring the open image area that utilizes discrete curvelet transform for separating the low‐intensity open area and the high‐intensity cell‐covered area. Then, a grey visible mask is created for cell‐free areas. The wound surface area and its change in time are calculated automatically by software.

### 2.10 Real‐time proliferation

The use of the xCELLigence® system enabled the observation of real‐time cell proliferation. In that experiment were used the E‐Plates (ACEA Biosciences), whose well bottoms are covered with gold microelectrodes. The test was started by measuring the background impedance of supplemented medium by placing them in the RTCA DP apparatus (ACEA Biosciences) and making the first measurement. Then, 10000 U‐118 MG cells were seeded on the same plate and incubated for 24 h under optimal growth conditions. From that moment on, until the end of the experiment, the system performed impedance measurements at 15‐min intervals. After 24 h, the cells were transfected with miR‐218 mimic, and measurements were continued for the next 48 h. The results are presented by the cell index unit. The normalization time point corresponds to the moment of transfection.

### 2.11 Thymidine incorporation assay

The cell culture was transfected and resumed for 20 h. Subsequently, a tritiated thymidine ([methyl‐3 H]‐thymidine)‐labelled solution with final radioactivity of 1 μCi per well was added for another four hours. To detach the cells, they were placed for 30 min at −80°C and then thawed at 37°C. The plate was placed in the MicroBeta FilterMate‐96 harvester, where the cells were transferred to the fibreglass filter paper Filtermat A (PerkinElmer) by three washes. Dried Filtermat A was placed in the plastic sample bags, and flooded with Betaplate Scint for Betaplete (PerkinElmer), then moved into the MicroBeta^2^ radiometric detector (PerkinElmer), which recorded the number of radioactive pulses per minute (counts per minute, cpm). As a positive control, cells treated with camptothecin (CPT) at a final concentration of 3 μM were used.

### 2.12 Real‐time adhesion

The xCELLigence® system together with the E‐Plates PET (ACEA Biosciences) was used. In each well of them, four rows of microelectrode sensors are removed, creating a window for cell visualization. Plates were covered with poly‐L‐lysine (Sigma‐Aldrich), incubated for one hour in 37°C and rinsed with phosphate‐buffered saline (PBS, VWR Life Science). Additionally, some wells were overlaid with 1% BSA for 20 min and acted as a negative control. An unsupplemented medium was analysed as a background. Then, 24 h earlier transfected cells were seeded 10,000 cells per well in serum‐free medium. Measurements took place every three minutes for four hours.

### 2.13 Single‐cell force spectroscopy (SCFS)

Cell deformability and adhesiveness were determined from the AFM measurements carried out in single‐cell force spectroscopy (SCFS) mode using CellHesion head (JPK Instruments). In SCFS, adhesion was quantified as a work of adhesion determined as an area under the part of the force curve corresponded to force/work needed to detach a single cell from surface. To prepare a cell force probe, the standard tipless cantilevers (Arrow‐TL, NanoWorld) characterized by nominal spring constant of 0.06 N/m were used. The average spring constant was 0.067 ± 0.016 N/m, as verified by the Sader method.^33^ First, bare cantilevers were cleaned and activated with an oxygen plasma for 2 min at the maximum power of 100 W (Diener Electronic GmbH, Zepto 1 device). Afterwards, cantilevers were immersed in 2 mg/ml concanavalin A (Con A, Sigma‐Aldrich) solution in PBS buffer (Sigma‐Aldrich) for 1 h and washed three times in PBS buffer. To use an individual cell as a force probe, the trypsinized solution of transfected cells was added to Petri dish (diameter 3.5 cm, Sarstedt) filled with DMEM with FBS, in which SCFS measurements were performed. Then, Con A‐functionalized cantilever was placed above a single cell and moved closer to its surface, followed by pressing it for about 5 s with the force of 5 nN. Afterwards, a slow cantilever withdrawing was applied until the cell fully detached from the surface. After 15–20 min of a pause time, the cell was usually attached to the cantilever surface. From this moment, the cell was used as a probe to collect force curves. For a single force probe, on average 5 force maps (scan size of 20 μm × 20 μm, on which a grid 6 pixels × 6 pixels was set) were recorded in randomly chosen locations on Petri dish surface. For a given sample type, 8–9 living cell force probes were used. For each sample type, on average 1800 individual force curves were recorded and analysed. The approach and retract speeds were kept at 8 μm/s. The measurement depth was 200 nm. As tipless cantilevers were used and the cell diameter was lower than the width of the cantilever, we treated our system like a single cell in between two fixed and compressive plates. Thus, from the approach part of the force curve, cell stiffness (N/m) was determined from a slope of the approach curve after contact with a cell surface (a linear regression was applied). Cell stiffness is calculated as a slope taking into account the range from the contact point to the maximum load force (maximum cantilever deflection). In parallel, from the retract part of the force curve, work of adhesion was calculated as an area under this part of force curves corresponding to adhesion using the methodology described elsewhere.^34^, ^35^

### 2.14 Cytoskeleton imaging

For structural cytoskeleton analysis, U‐118 MG cells were cultured on microscope coverslips and transfected under standard conditions as described previously. 24 h post‐transfection, cells were fixed with the use of Image‐iT™ Fixation/Permeabilization Kit (Invitrogen) according to the manufacturer’s protocol. F‐actin fibres were visualized by phalloidin conjugated to tetramethylrhodamine (Invitrogen) with simultaneous use of DAPI (Sigma‐Aldrich) to visualize cell nuclei. Staining was performed according to the manufacturer’s protocol. Pictures were obtained with the use of Leica TCS SP5 confocal microscope and software LAS X SP8 (Leica).

### 2.15 Gene expression analysis based on the Gliovis database

TN‐C and SDC‐2 gene expression analysis for large groups of patients was performed using the Gliovis online tool (gliovis.bioinfo.cnio.es). In order to comprehensively present the expression of these genes in large group of tissues, databases from two independent projects (TCGA and Rembrandt) were used. Data set HG‐U133A contained information from 10 non‐tumour samples and 528 samples described as GBM. The Agilent‐4502A data set contained the same number of healthy samples and 489 samples of glioblastoma. The Rembrandt database, on the contrary, has a set of 28 healthy samples, 225 described as non‐tumour glioma and 219 GBM samples. Only the “non‐tumour” and “GBM” data sets were used from the Rembrandt database.

### 2.16 Statistical analysis

The results are presented as a mean value ± standard deviation (SD). They were averaged depending on the applied methods. For AFM measurements, averaging was performed for 8–9 cell force probes. For other experiments, 3 biological replicates were applied. The statistical significance of the obtained results was evaluated using the Open Office Calc ver. 4.1.1 (Apache) and GraphPad Prism ver. 5.1 (GraphPad Software). Differences between the mean values of the test and the control samples were evaluated using anova variance extended by the Tukey or the Bonferroni post hoc tests. Statistically significant results were assigned as: * for *p* < .05; ** for *p* < .01; and *** for *p* < .001; no statistical significance was found for *p* ≥ .05.

## 3 RESULTS

### 3.1 In glioblastoma, the expression level of miR‐218 correlates inversely with the expression levels of the ECM components TN‐C and SDC‐2

**FIGURE 1:**
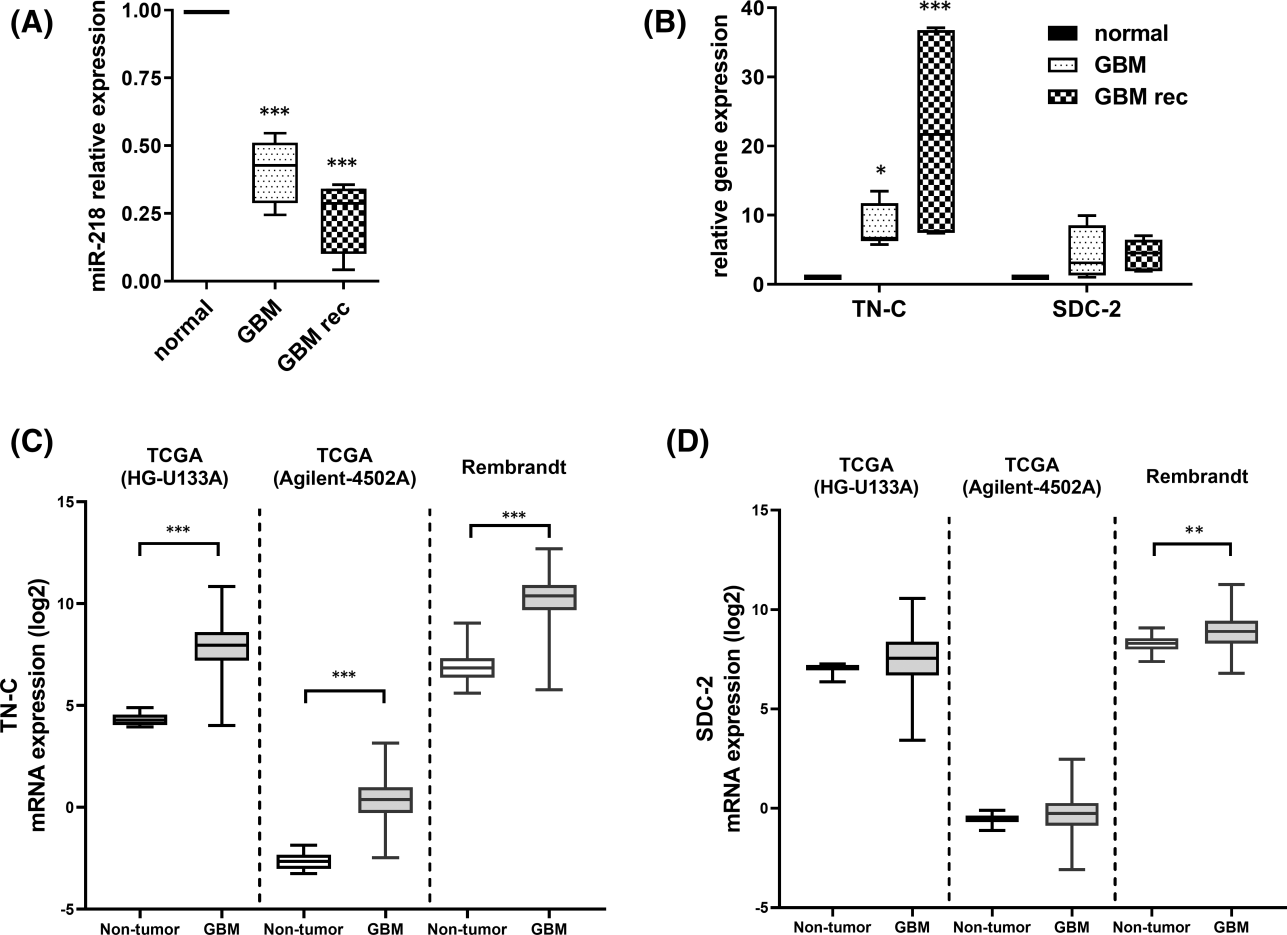
miR‐218 expression in primary (GBM) and recurrent glioblastoma (GBM rec) tissues and its putative target mRNAs. (A) qRT‐PCR analysis of GBM (*n* = 10) and GBM rec samples (*n* = 9) in comparison with a healthy brain RNA sample (*n* = 1). Healthy brain sample consists of RNA pooled from 23 donors. Data are shown as the mean ± SD values. One‐way anova and the post hoc Bonferroni test, ***p < .001. (B) qRT‐PCR analysis of the tenascin‐C and syndecan‐2 mRNA expression levels in GBM and GBM rec tissues in comparison with RNA from healthy brain RNA sample (*n* = 1). Healthy brain sample consists of RNA pooled from 23 people Data are shown as the mean ± SD values. Mixed‐model analysis and the post hoc Bonferroni test; *p < .05 and ***p < .001. (C,D) Expression of TN‐C and SDC‐2 in GBM tumour from TCGA and Rembrandt databases examined using the Gliovis database. Tukey's test, ***p < .001 and **p < .01

Our previous study revealed that there were 97 miRNAs differentially expressed in glioblastoma compared with those in the healthy brain.^36^ Forty‐one of these miRNAs showed a reduced expression level in malignant gliomas. Among these miRNAs, we found miR‐218 to be significantly downregulated in brain tumour tissues. We further confirmed the expression level of miR‐218 in primary and recurrent GBM via qRT‐PCR analysis (Figure 1A). The levels of miR‐218 expression in primary tumour tissue and recurrent GBM tissue were 56% and 69% lower, respectively, than those in healthy brain tissue.

Given the profound downregulation of miR‐218 in GBM, we sought to investigate its putative targets. To identify the binding sites in the 3′UTRs of genes that can be potentially regulated by selected miRNAs, we used prediction software such as ENCORI, miRDB, PicTar and TargetScan. Interestingly, among the predicted targets, we found several genes encoding ECM proteins, such as tenascin‐C (TN‐C), syndecan‐2 (SDC‐2), attractin (ATRN), cadherin‐2 (CDH‐2), cadherin‐8 (CDH‐8), extracellular leucine‐rich repeat and fibronectin type III domain containing 2 (ELFN2), fibronectin leucine‐rich transmembrane protein 2 (FLRT2), hyaluronan and proteoglycan link protein 1 (HAPLN1), 5‐hydroxytryptamine receptor 7 (HTR7), neurocan (NCAN), proteoglycan (PRG4), reelin (RELN) and sarcoglycan zeta (SGCZ) (Supplementary information 1).

### 3.2 TN‐C and SDC‐2 are direct targets of miR‐218

Highly ranked binding targets of miR‐218 were subjected to further analysis. We focused specifically on TN‐C and SDC‐2 and investigated their gene expression levels via qRT‐PCR. The tenascin‐C level was significantly increased in all examined tumour samples—that is eightfold higher in primary tumour tissue and 21‐fold higher in recurrent tumour tissue than in healthy brain tissue. In the case of SDC‐2, our analysis indicated an increase of approximately fourfold in GBM (Figure 1B). Our qPCR analysis is additionally supported by the data from large data sets coming from the sequencing of glioblastoma multiforme deposited in TCGA and Rembrandt databases. The expression profile of TN‐C and SDC‐2 in our experiment is comparable to the expression profile of aforementioned genes in databases (Figure 1C,D). There is a definite difference in TN‐C expression levels between normal and tumorous tissue based on the database analysis, what is fully in line with our research and databases. Analogously, the results of our research and the values obtained from databases indicate a statistically significant distinction in the level of TN‐C expression between normal tissue and GBM. In the case of SDC‐2, differences with a statistical value were observed only in the Rembrandt database.

**FIGURE 2:**
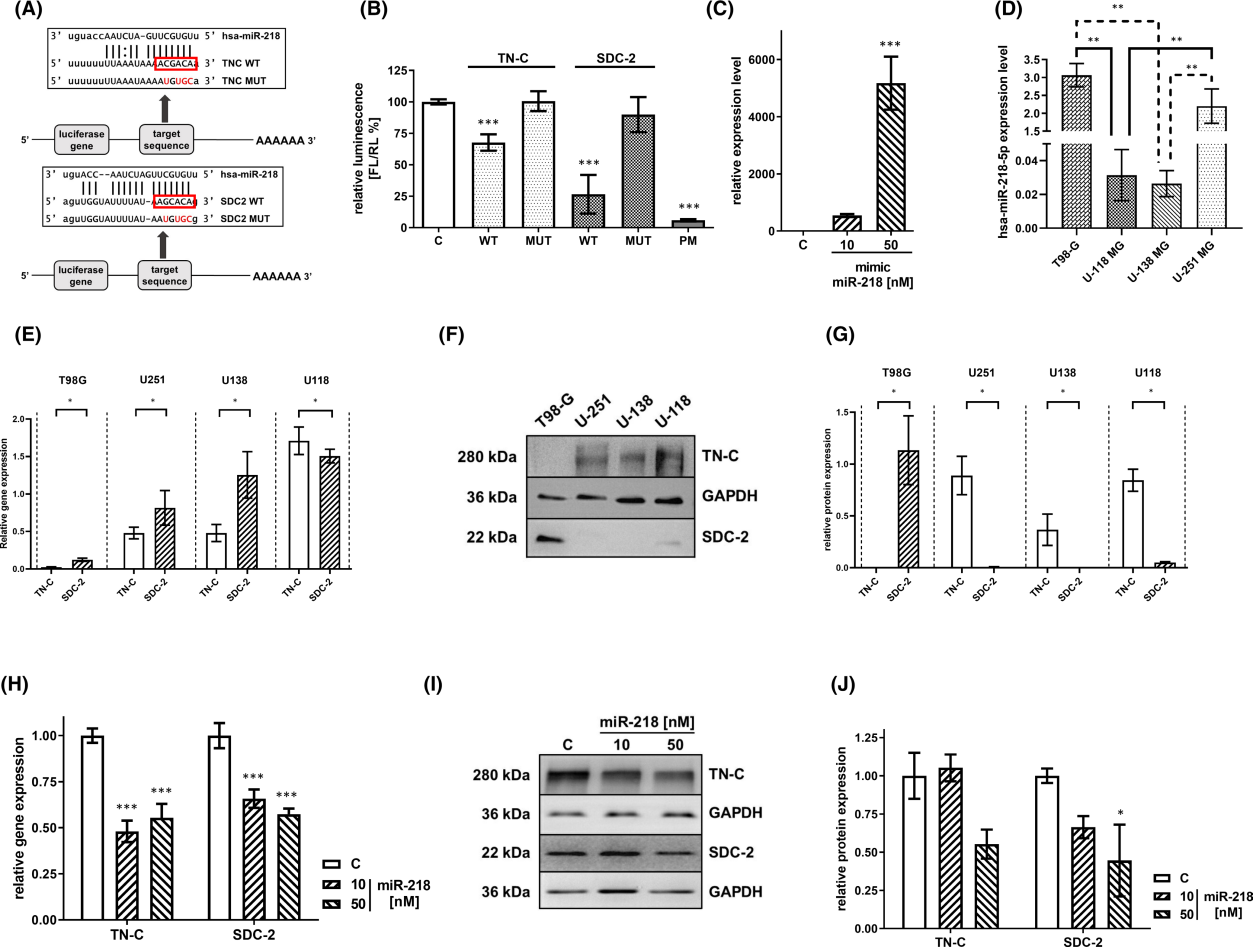
Regulation of TN‐C and SDC‐2 by miR‐218. (A) Schematic representation of the interaction between miR‐218 and 3′UTRs of its targets. The seed region is enclosed in a red box. The putative conserved sequences in the SDC‐2 and TN‐C targets are denoted as the wild type (WT). The non‐conserved nucleotides within the seed region of the mutant 3′UTRs are marked in red in the construct named “mutant” (MUT). (B) Relative repression of luciferase expression. Reporter constructs carrying a single binding site were tested. miR‐218 activity in 5 constructs was measured in parallel (Control U‐118 MG cells—C, WT, MUT and perfect match—PT as a positive control in the experiment). Data are shown as the mean ± SD values. ***p < .001. (C) Overexpression of miR‐218 as a result of miR‐218 mimic transfection, as evaluated by qRT‐PCR. The measured expression level of TN‐C and SDC‐2 in different glioma cell lines with the use of qRT‐PCR. (D) Expression level of miR‐218 in T98‐G, U‐118 MG, U‐138 MG and U‐251 MG cell lines. Data are shown as the mean ± SD values. **p < .01 (E) and Western blot (F,G). All cell lines were cultured in corresponding cell culture media in the same period of time, and materials for analysis were isolated in the same batch to avoid unnecessary variability. (H) The quantified effects of transfection of U‐118 MG cells with the miR‐218 mimic at 10 nM and 50 nM concentrations on mRNA levels, as measured by qRT‐PCR, and on protein levels, as established by Western blot analysis (I,J). Cells transfected with scrambled siRNA were used as the control—(C). Data are shown as the mean ± SD values. Two‐way anova and the post hoc Bonferroni test, *p < .05 and ***p < .001

All algorithms used for miR‐218 target prediction showed one binding site within the 3′UTR of both the TN‐C and SDC‐2 mRNAs. We employed a set of reporter constructs in a luciferase assay to experimentally verify the predicted binding of miR‐218 to its target sites within the 3′UTRs of TN‐C and SDC‐2. The following constructs were tested in parallel: wild‐type reporters (WT) containing a single native binding site for either miR‐218, constructs with mutations (MUT) disrupting the 5′ seed site (negative controls) and constructs with perfect complementarity (PM) to the miR‐218 binding site (positive controls) (Figure 2A). Considering our previous analysis revealing the inverse correlation between miR‐218, TN‐C and SDC‐2 expression, we validated the predicted miRNA–mRNA interactions using a miRNA overexpression system. Specifically, U‐118 MG cells were co‐transfected with reporter constructs and miRNA‐encoding plasmids. Co‐transfection experiments showed that cells transfected with miR‐218 had significantly inhibited luciferase activity compared to cells transfected with negative control (MUT) miRNA (Figure 2B). The reduction in luciferase activity was reproducible and statistically significant for both WT constructs, with suppression of 33% and 74% for TN‐C and SDC‐2, respectively. miR‐218 did not inhibit the luciferase activity of reporter vectors containing the TN‐C and SDC‐2 3′UTRs with mutations in the putative miR‐218 binding site. This study provides evidence of the direct binding of miR‐218 to the TN‐C and SDC‐2 3′UTRs and positively validates this miRNA as a negative regulator of these ECM molecules.

### 3.3 miR‐218 regulates TN‐C and SDC‐2 protein levels

We sought to determine the role of miR‐218 in the regulation of TN‐C and SDC‐2 at the protein level in GBM cells by a gain‐of‐function approach. We transfected U‐118 MG cells with synthetic miRNA (miRNA mimic) at concentrations of 10 and 50 nM. The final miR‐218 mimic concentration of 10 nM boosted the expression of miR‐218 by almost 500‐fold compared with the control level, while 50 nM increased the expression by more than 5000‐fold (Figure 2C).

In order to select an appropriate research model, we evaluated expression levels of miR‐218, TN‐C and SDC‐2 in four glioblastoma cell lines: T98‐G, U‐118 MG, U‐138 MG and U‐251 MG. The U‐118 MG and U‐138 MG lines represented the lowest miR‐218 expression level, with no statistical differences between them (Figure 2D). TN‐C was the most expressed on both mRNA and protein levels in U‐118 MG cell line, while in T98‐G, it was undetectable (Figure 2E). SDC‐2 in Western blot analysis was under detection level for U‐138 MG and U‐251 MG cell lines. qRT‐PCR revealed the highest expression of SDC‐2 in U‐118 MG in relation to other cell lines. We found contradictory results between the levels of SDC‐2 mRNA and protein in T98‐G (Figure 2F,G). Summarizing our analyses, we selected the U‐118 MG line for further research, taking into account its low expression level of miR‐218, high level of TN‐C and possible detection of SDC‐2.

To further verify the function of miR‐218 and its impact on TN‐C and SDC‐2 expression levels, we performed analyses at both the mRNA and protein levels by qRT‐PCR and Western blot, respectively. At the mRNA level, transfection with the miR‐218 mimic in two concentrations, 10 nM and 50 nM, resulted in a reduction in the tenascin‐C expression level of 45%–52% in comparison with the control level. In the case of syndecan‐2, we observed a decrease of 34% for 10 nM and 43% for 50 nM of mimic miR‐218 (Figure 2H). Western blot analysis revealed downregulation of SDC2‐2 expression by 34–55%. For TN‐C, protein level after miR‐218 mimic 10 nM supplementation increased by 5% and decreased by 45% after miR‐218 mimic 50 nM transfection (Figure 2I,J).

### 3.4 miR‐218 affects the ECM composition

**FIGURE 3:**
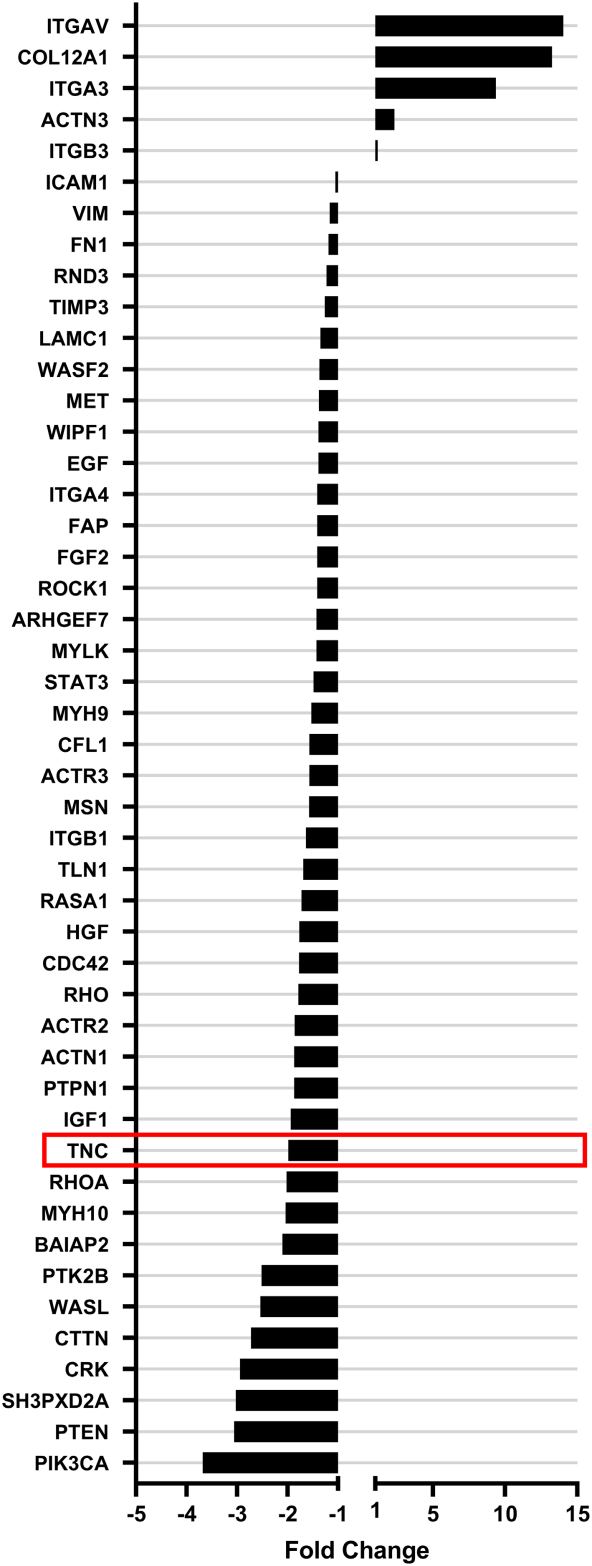
Cluster analysis of mRNAs encoding ECM components that were differentially regulated in the GBM cell line after miR‐218 transfection. The quantified effects of transfection of U‐118 MG cells with the miR‐218 mimic at a 50 nM concentration on the expression levels of genes, as determined by qRT‐PCR of a Human Cell Motility and Extracellular Matrix & Adhesion Molecules RT2 Profiler PCR Array. Cells transfected with scrambled siRNA were used as the control

Given the above results, we have evaluated the miR‐218 overexpression on the ECM composition. To test this hypothesis, we used a Human Cell Motility and Extracellular Matrix & Adhesion Molecules RT^2^ Profiler PCR Array and profiled the expression of *n* = 160 genes related to the motility and adhesion pathways (Figure 3). More than 95% of the transcripts were detected, but the expression of CDH1, ANOS1, CNTN1 and MMP8 was not detected by this technique in our analysis (data not shown). Quality control parameters (positive PCR controls and reverse transcription controls) showed good reproducibility and efficiency with the web‐based RT^2^ profiler PCR Array Data Analysis program. In this paper, we include results where *p*‐value is lower than .05 and fold change value is in the range (∞,–1) ∪ (1,∞). A full set of data obtained from RT^2^ profiler plates is included in supplementary materials (Supplementary information 2). Thus, we identified 47 genes displaying significantly different expression as a result of miR‐218 overexpression (Supplementary information 3). It became evident that miR‐218 overexpression led mostly to decrease in the expression of genes, among which were tenascin‐C, as its direct ECM target, as well as the other genes involved in cytoskeletal reorganization. We observed the 1,96‐fold reduction in the TN‐C expression level. This result is in line with our real‐time PCR analyses described above, in which we obtain also almost twofold reduction in TN‐C expression level after miR‐218 mimic 50 nM transfection. We further hypothesized that changes in the ECM composition due to miR‐218 overexpression also affect the mechanobiological properties of cancer cells.

### 3.5 Impaired cell migration after miR‐218 treatment

**FIGURE 4:**
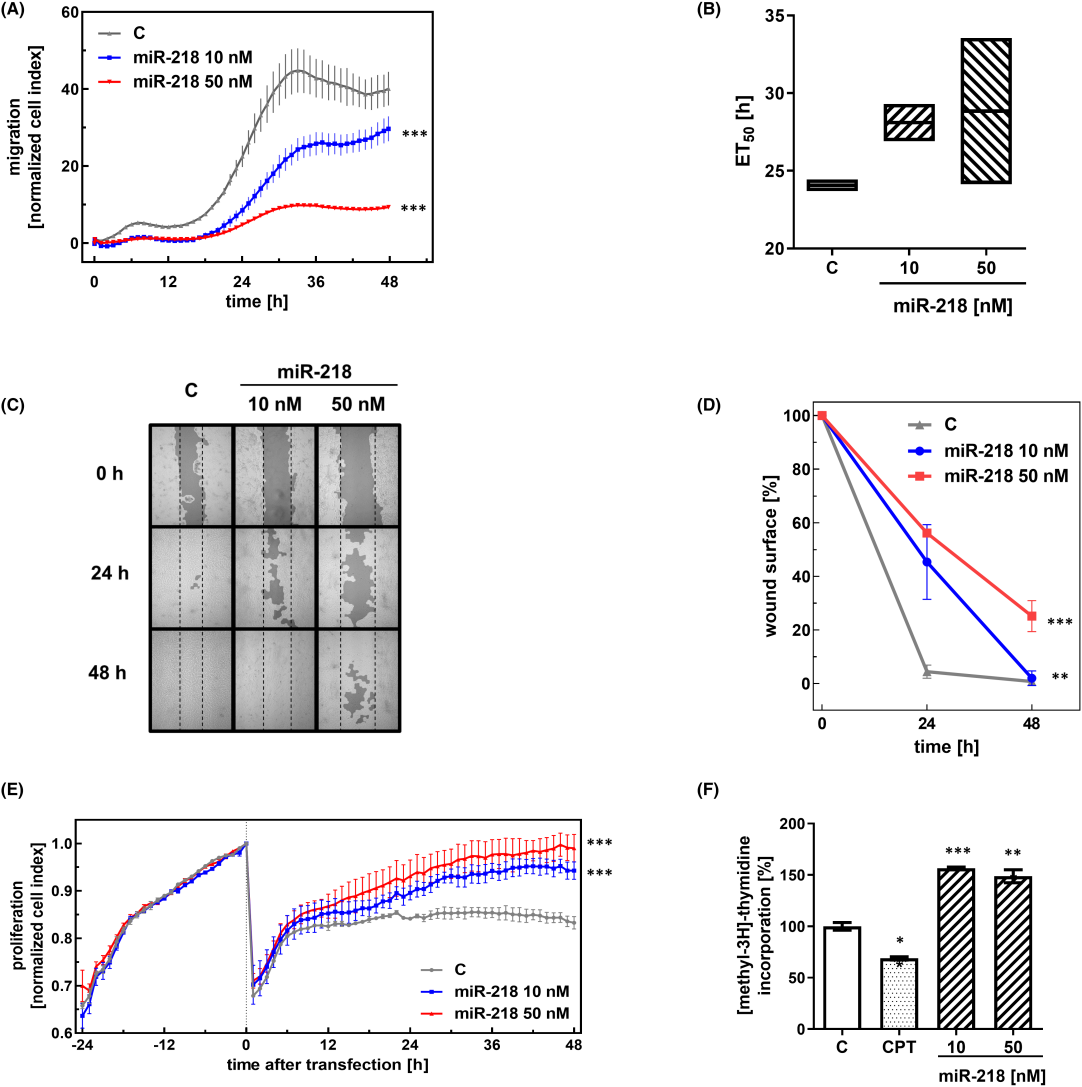
Effect of miR‐218 on the migration and proliferation of glioblastoma cells. (A) The migration of U‐118 MG cancer cells was studied using an xCELLigence system. Cells in serum‐depleted medium were transfected with the miR‐218 mimic (10 and 50 nM). Control (C)—cells treated with scrambled siRNA. Data are shown as the mean ± SD values. One‐way anova and the post hoc Bonferroni test, ***p < .001. (B) The half‐maximal effective time (ET50) was calculated for each miR‐218 concentration to generate dose–response curves. The ET50 values were normalized to control (C) cells treated with scrambled siRNA and plotted as the normalized ET50 of cell migration against the miR‐218 concentration. (C) The wound healing assay after miR‐218 mimic transfection. The dark grey areas indicate the surface area of the wound. (D) The calculation of the wound area (%) 24 and 48 h post‐transfection. Control U‐118 MG cells (C) were treated with scrambled siRNA. Data are shown as the mean ± SD values. One‐way anova and the post hoc Bonferroni test, **p < .01 and ***p < .001. (E) Proliferation of U‐118 MG cancer cells analysed with the xCELLigence system. Cells were transfected with the miR‐218 mimic (10 and 50 nM). Control (C)—cells treated with scrambled siRNA. Data are shown as the mean ± SD values. One‐way anova and the post hoc Bonferroni test, *p < .05 and **p < .01. (F) The thymidine incorporation assay on miR‐218 mimic‐transfected cells. As the positive control, cells treated with camptothecin were used. One‐way anova and post hoc Bonferroni test, *p < .05, **p < .01 and ***p < .001

To explore the impact of miR‐218 on cell migration, we compared the migration rate of miR‐218‐transfected U‐118 MG cells with that of non‐treated (negative control) cells (Figure 4A). The mathematical interpretation of the impedance (CI value) for each experimental condition was recorded over time and fitted to a four‐parameter logistic non‐linear regression model (Figure 4B). Transfection of 10 and 50 nM miR‐218 increased the ET_50_ by an average of 4 and 4.7 h, respectively.

As the second independent experiment, we carried out a wound healing assay. 48 h after transfection, the largest unhealed area was observed in the miR‐218 50 nM sample and accounted for 25.2% of the original wound area, while in the control sample, it was 0.8% (Figure 4C,D). At a concentration of 10 nM, the unhealed area was 2% of the original wound area. The most pronounced difference in the function of miR‐218 was revealed at the 24‐h time point, when the wound areas in the control and mimic 10 nM and mimic 50 nM samples were 4.4%, 45.3% and 56.2%, respectively. Both experiments indicated a delay in GBM cell migration rate upon miR‐218 treatment.

We analysed further the real‐time cell proliferation with the xCELLigence system. The graph shows the raw experimental data presented as the dependence of the cell index unit used in the xCELLigence system on the time (Figure 4E). In this way, the time course of proliferation changes with overexpression of miR‐218 is illustrated. At the point on the timeline corresponding to 24 h, the curve inflection indicates the time of transfection. The stimulating effects on proliferation are seen at the final points of the curves, 48 h post‐transfection. We observed an increase in the cell index by 14% at a miR‐218 mimic concentration of 10 nM and by as much as 19% at 50 nM. This demonstrates the directly proportional relationship between the increase in the proliferation rate and the expression of miR‐218.

A [methyl‐3H]‐thymidine incorporation assay was performed to complement the proliferation analysis with the xCELLigence system. In this study, the degree of incorporation of radioactively labelled thymidine was evaluated and translated into the replication potential of cells. In addition to the standard trials used, we analysed the effect of 3 μM camptothecin, which has a confirmed pro‐apoptotic effect,^37^ as a positive control in the experiment. The incorporation rate in CPT‐treated cells was 69% compared with that in control cells. miR‐218 mimic transfection increased the incorporation of tritiated thymidine by 57% at 10 nM and 49% at 50 nM compared with that in control cells (Figure 4F).

### 3.6 miR‐218 enhances glioma cell adhesion

**FIGURE 5:**
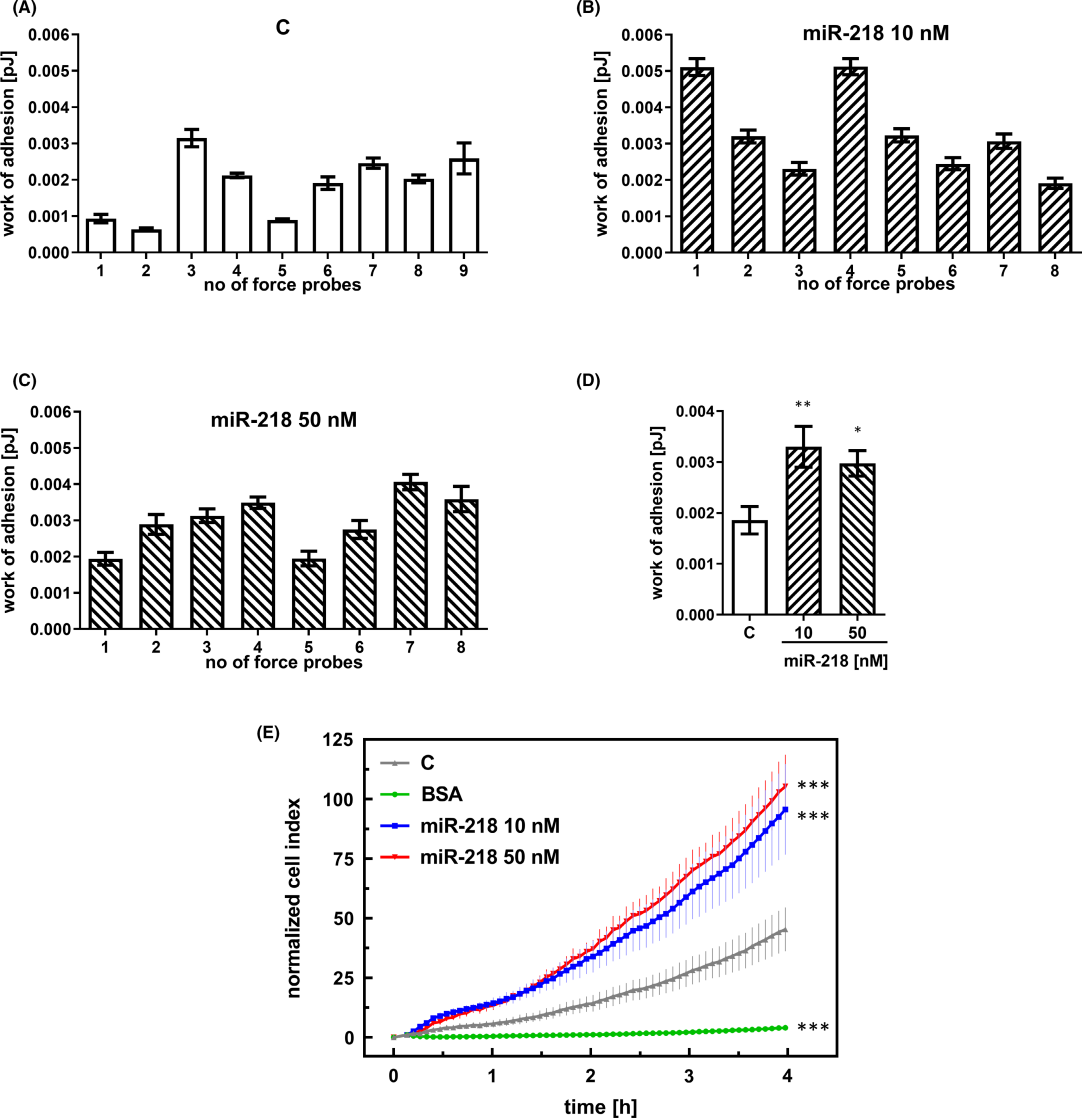
Adhesion of GBM cells increases after miR‐218 treatment. The adhesive properties of U‐118 MG cells were quantified by SCFS using single cells as force probes. Data for control, treated with scrambled siRNA cells (A), cells transfected with the miR‐218 mimic at concentrations of 10 nM (B) and 50 nM (C), and the average result over all measurements (D). (E) Real‐time adhesion measured with the xCELLigence system. The graph shows the final impedance values minus the initial values for the corresponding samples. Control (C)—cells treated with scrambled siRNA. Cells suspended in bovine serum albumin (BSA) were used as the positive control. Data are shown as the mean ± SD values. One‐way anova and the post hoc Bonferroni test, *p < .05, **p < .01 and ***p < .001

To explore the impact of miR‐218 on U118‐MG cells, the cell surface properties were quantified using AFM in SCFS mode (Figure 5D). These properties are quantified by calculating the work of adhesion, which is defined as the work required to detach a single cell from the surface. In this scenario, each single cell was used as a force probe. For control cells, the work of adhesion ranged from 0.00064 pJ to 0.00315 pJ, with a mean ± standard deviation of 0.00186 ± 0.00081 pJ (Figure 5A). The analogous variability in cell adhesion after miR‐218 treatment ranged from 0.00191 pJ to 0.00512 pJ (mean ± standard deviation = 0.0033 ± 0.00113 pJ) and from 0.00194 pJ to 0.00406 pJ (mean ± standard deviation = 0.00297 ± 0.0007 pJ) for concentrations of 10 nM and 50 nM, respectively (Figure 5B,C). Real‐time adhesion measurements performed with the xCELLigence system showed changes in the attachment of cells to the plate surface during the observation period (Figure 5E). The cell index of adhesion for cells treated with 10 nM miR‐218 mimic was 2.5‐fold greater than that of control cells. In the case of 50 nM miR‐218 treatment, the observed index was 3 times higher than that measured in the control cells. From the beginning of the experiment to its end, the trends in the particular samples did not change. We assumed that the observed changes were miR‐218‐dependent, since the negative control cells did not bind to the plate covered with BSA. The untreated cells also showed low adherence compared with the miR‐218‐treated cells.

Thus, regardless of the technique used for the adhesion study, these two independent experiments demonstrated an increase in the adhesion of GBM cells treated with the miR‐218 mimic.

### 3.7 Overexpression of miR‐218 impacts cell stiffness

**FIGURE 6:**
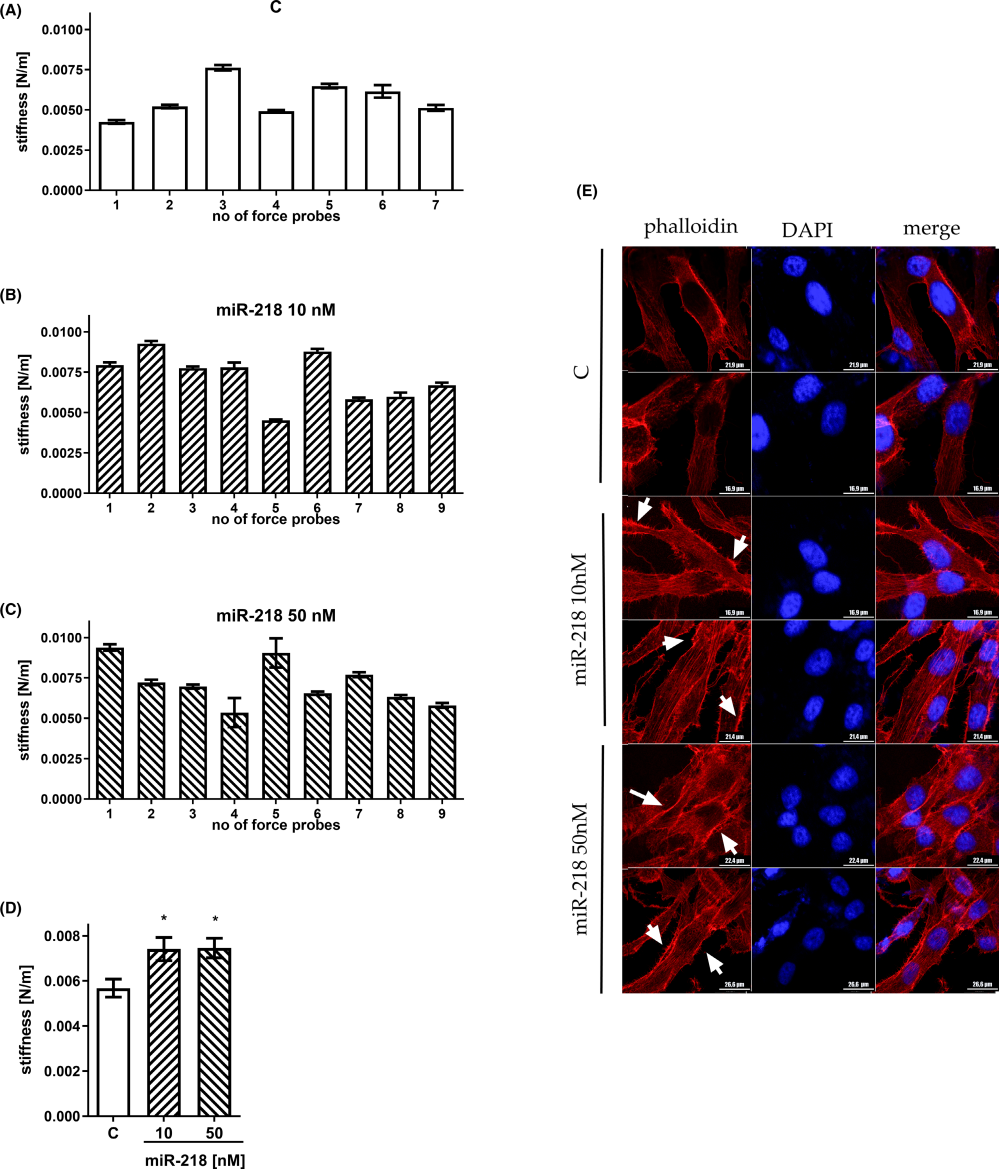
Mechanical properties of GBM cells after miR‐218 treatment. Stiffness of U‐118 MG cells quantified based on AFM elasticity measurements and expressed in N/m. Data for control, treated with scrambled siRNA cells (A), cells transfected with the miR‐218 mimic at a 10 nM concentration (B) and a 50 nM concentration (C), and the average result over all measurements (D). (E) Confocal imaging of the actin cortex. Phalloidin (red) staining and DAPI (blue) staining were performed to visualize actin fibres and cell nuclei, respectively. Z‐stack images were acquired. White arrows point to visible changes in actin structures. Data are shown as the mean ± SD values. One‐way anova and the post hoc Bonferroni test, *p < .05

Most surface receptors are linked not only to ECM proteins but also to actin filaments forming the actin cortex.^38^ Thus, in our next step, we verified whether changes in GBM cell adhesive properties contribute to the overall mechanical properties of these cells. Cell stiffness was measured for cells compressed between the surface and a tipless cantilever; therefore, it was calculated as the slope of the approach part of the recorded force curves and expressed in N/m (Figure 6D).

For all sample types, that is control cells or cells treated either with 10 nM or with 50 nM miR‐218, the stiffness of compressed cells remained mildly changed. Here, a smaller variability in mechanical force was observed for a given cellular force probe, as indicated by the standard deviation values. For control cells, the stiffness varied from 0.00425 N/m to 0.00763 N/m with a mean ± standard deviation of 0.00568 ± 0.00106 N/m (Figure 6A). Cells treated with miR‐218 were characterized by a stiffness ranging from 0.00451 N/m to 0.00927 N/m (mean ± standard deviation = 0.00741 ± 0.00155 N/m) and from 0.00535 N/m to 0.00938 N/m (mean ± standard deviation = 0.00746 ± 0.00130 N/m) for concentrations of 10 nM and 50 nM, respectively (Figure 6B,C). Regardless of the final values of cell stiffness, an increasing trend was seen. For cells treated with 10 nM miR‐218 mimic, the stiffness was increased by 30%, and for cells treated with 50 nM miR‐218 mimic, the increase was 31% (Figure 6D). As the cell stiffness measured using AFM is related to the organization of the actin cytoskeleton, it was further visualized using fluorescently labelled F‐actin to verify the effect (Figure 6E). Fluorescence images of the actin cortex show differences between control and miR‐218‐treated cells. Cells treated with miR‐218 showed a higher level of actin filament organization. In these cells, the actin filaments became organized more horizontally along the long axis of the cell compared with those in control cells, where they were more dispersed. After treatment with 50 nM miR‐218, also small changes in actin structures appeared at the cell surface. The F‐actin dynamics and changes in filament organization directly support the increased stiffness of cells treated with the miR‐218 mimic. The observed changes confirm then the hypothesis that cell stiffness is related to the cytoskeleton.

## 4 DISCUSSION

The malignancy of glioblastoma depends on its ability to infiltrate adjacent tissues and to create secondary lesions.^39^ The aggressive growth of glioma tumours and difficulties in developing an effective treatment scheme have led to intense integration of medical and molecular biological research. Remodelling of the ECM and miRNA deregulation are known processes contributing to GBM cell invasion and brain infiltration.^40^, ^41^, ^42^ In this study, we show that miR‐218 can play a role in regulation of ECM remodelling in glioblastoma cell line and could be investigated further as a possible important regulator of GBM. We found that TN‐C and SDC‐2 are directly regulated by miR‐218, resulting in alterations in the ECM composition and changes in the mechanical properties of the cells. Although our previous finding identified miR‐218 as a potential tumour suppressor in GBM,^36^ the mechanism of miR‐218 action in GBM is still poorly understood. The sequence of miR‐218 is located within intron 15 of the SLIT2 gene, in which promoter region CpG island is hypermethylated in GBM.^43^, ^44^ The positive correlation between SLIT2 and miR‐218 expression has been shown, what indicates that these two molecules are transcribed together.^45^ The SLIT2 downregulation in GBM in consequence leads to the further decreased expression of miR‐218.^46^ Moreover, the expression level of miR‐218 in GBM might be invoked by the feedback mechanism. The decreased expression of miR‐218 can directly increase the expression of effector molecules such as RSK2, 6SK1 and PDGFRα, maintaining then the activity of the RTK pathway at a high level. RTK‐conducted signals stimulate the expression of the STAT3 gene, whose product together with BCLAF1 binds directly to the miR‐218 locus, thereby suppressing its expression.^47^ Our previous finding confirmed then the decreased expression of miR‐218 both in primary and in recurrent tumours by 50% and 70%, respectively. Decreased miR‐218‐5p expression levels have also been reported in other types of human cancer, such as medulloblastoma, thyroid cancer and cervical cancer.^48^, ^49^, ^50^ We confirmed that the predicted miR‐218 targets, the ECM components TN‐C and SDC‐2, are directly regulated by miR‐218. We used a dual‐luciferase assay and miR‐218 mimic to verify these functional interactions. The effects were detectable at both the mRNA and protein levels for both TN‐C and SDC‐2. Proteins derived from these transcripts are potentially key factors in the ECM of cancer cells.^15^, ^21^ The presence of TN‐C in cancer tissues was initially considered as a characteristic feature of only gliomas,^51^ with its expression increasing in proportion to the degree of brain tumour malignancy.^52^ Its presence was found to increase the proliferation and invasiveness of cancer cells and to take part in the process of angiogenesis.^53^ The role of TN‐C in the neoplastic process is to reduce the adherence of cells, leading to the spread of the tumour. On the surface of healthy fibroblasts, fibronectin (FN) interacts with transmembrane proteins—integrins and syndecan‐4 (SDC‐4). The Rho protein is activated, and the properties of actin filaments are changed, resulting in cell adhesion. In pathological conditions, tenascin‐C blocks the interaction between FN and SDC‐4. The Rho protein is not activated, resulting in a lack of cell adhesion signals.^54^ Considering the impact of TN‐C on cancer cells and its apparent overexpression in glioblastoma tissues, it could be considered as a promising therapeutic target. We have already shown that the treatment with a double‐stranded RNA targeting TN‐C increased the average survival rate of patients.^55^

An increased level of syndecan‐2 is a characteristic of actively migrating cells.^56^ Overexpression of this protein in melanoma cells indirectly contributes to an increase in the level of FAK kinase phosphorylation, which has a positive impact on the migration capability of these cells.^17^ In lung cancer, SDC‐2 deficiency prevents cells from adhering to FN, which blocks their migration.^57^

ECM has become one of the most important focuses of cancer research, as it was shown to play a major role in the development of metastasis.^58^ Pronounced ECM remodelling affects the invasion and migration of cancer cells.^59^, ^60^ The mechanical properties of the ECM have an impact on fibronectin fibril assembly, cytoskeletal stiffness and the strength of integrin–cytoskeleton linkages, the factors found to be important for cell motility, and thus also on adhesive properties.^61^ As demonstrated in previous reports, a more rigid ECM promotes glioma cell migration.^62^ On highly rigid ECMs, tumour cells spread extensively, form prominent stress fibres and mature focal adhesions, and migrate rapidly.^62^ Our results are in line with these observations, as we showed a decreased cell migration rate after mir‐218 overexpression, with subsequent downregulation of TN‐C expression. These direct effects were enhanced by the indirect effect of miR‐218 on a number of proteins, for example fibronectin, collagens or laminins. Thus, with miR‐218 overexpression, we observed changes in the ECM leading to slowed cell migration, most likely induced by changes in overall ECM rigidity.

The obtained data revealed the decrease in the rate of cell migration upon the overexpression of miR‐218, but at the same time also an increase in their proliferation potential (Figure 4). Our observations seem to be consistent with the “go‐or‐grow” hypothesis, according to which the division of neoplastic cells and their movement are two temporally exclusive events.^63^ The “go‐or‐grow” decision is strictly regulated and modulated by changes in the tumour microenvironment, which allows cells to “go” towards more favourable conditions to proliferate at the distant site or to “grow” and to stay at the site of origin, if their current environment provides the proper conditions for tumour growth. Changes in miRNA expression, followed by the ECM remodelling, can modulate the “go‐or‐grow” decision. As it has already been shown previously, the considerable overexpression of miR‐9 in glioma cells inhibits proliferation but concurrently promotes migration.^64^ Evidence indicates that mechanical properties and deformability can also be used as biomarkers to distinguish between healthy and cancer cells. The deformability of a whole cell, which depends on the properties of the cytoplasm, the cytoskeleton and the nucleus, can be defined in terms of the response of the cell to an applied stress. One of the techniques that enables the measurement of biophysical properties of cells, such as adhesion and stiffness, is AFM.^65^ We evaluated the mechanobiological properties of GBM cells, including adhesion and stiffness, upon miR‐218 mimic treatment. We obtained real‐time measurements in cell culture (xCELLigence system) and measured physical forces and the work of adhesion^66^ by application of AFM in SCFS mode. This approach allowed us to quantify the adhesion of single cells. SCFS analysis revealed strengthened adhesion of GBM cells upon miR‐218 overexpression, hence indicating the direct connection between miR‐218 and ECM component regulation.

GBM cells, similar to other solid cancers, can remodel the surrounding microenvironment from a normal brain to a stiffer tumour microenvironment through the combination of proteolytic degradation of some ECM components and secretion of other novel ECM components.^67^ In our analysis, the stiffness of miR‐218‐transfected cells as measured by AFM was 30% higher than that of the control cells. Despite the variability observed in the experiment, a clear difference was observed, as the overall stiffness was measured to increase in cells treated with miR‐218. The differences observed in the experimental cell group might have stemmed from the distributed contribution of surface receptors on an individual single cell, which can thus impact the adhesion of that cell.^68^, ^69^ It has already been shown that tumours can become stiffer than normal tissues due to increased Rho‐dependent cytoskeletal pressure, generating excessive growth, focal adhesions, adjacent joint division and tissue disruption.^70^ Stiffness also directly depends on the malignancy of the tumour. It is known that invasive GBM tumours produce stiffness‐promoting factors such as collagen, fibronectin and laminins, which may suggest that the production of these proteins is disrupted after miR‐218 overexpression.^71^

An increase in stiffness has also been observed in many different types of cancer cells, such as breast cancer, melanoma, prostate cancer and cervical cancer cells. An important aspect of cell stiffness is the ratio of cancer to normal cells. While cancer cells are less stiff than normal cells,^72^ the same pattern of stiffness is also observed in malignant versus non‐malignant tissues in breast cancer,^73^ bladder cancer^74^ and prostate cancer.^75^ In our research, glioblastoma cells with miR‐218 overexpression were approximately 30% stiffer than non‐treated cells. Increased stiffness in brain tissues can be correlated with diseases such as brain abscess or with cytoskeletal maturation in brain cells.^76^ The correlation of cytoskeletal maturation with an increase in cell stiffness has been observed for astrocytes, in which the AFM‐measured stiffness may increase sevenfold in a 5‐week observation period during development.^77^ In miR‐218‐treated GBM cells, the actin cytoskeleton was slightly rearranged, which could explain the increase in cell stiffness.

The minor discrepancy in the relation between cell surface adhesive properties and cell stiffness measured in our study can be explained by the different scales of measurements. For cell adhesion, SCFS measurements are limited to local changes occurring on the cell surface, while the stiffness reflects the overall mechanical properties of cells. Thus, we analysed stiffness assuming an indentation depth of 200 nm. This value assures the sensing of a superficial layer of actin filaments. Additionally, brain tissue is much softer than other tissues. The value of Young’s modulus ranges from 1 to 1.9 kPa for white matter and from 0.8 to 1.4 kPa for grey matter, depending on the measurement technique.^78^, ^79^ Independent methods, that is SCFS and the xCELLigence system, showed similar increases in the adhesive properties of U‐118 MG cells upon miR‐218 treatment. Collectively, these results demonstrated that miRNA‐218 strongly affects the expression of genes encoding cell surface receptors responsible for the adhesive properties of cells.

We also found that miR‐218‐treated cells are more rigid than non‐treated cells, which presumably might prevent them from undergoing extravasation and intravasation during migration and invasion events. We thus hypothesized that miR‐218 overexpression can support the maintenance of the non‐invasive cell phenotype, which is correlated with differences in mechanical properties. The observation is more important when one realizes the importance of ECM rigidity in the perivascular space. It has already been shown that this part of the brain tissue is more rigid in GBM, thus promoting glioma cell migration.^62^

As we have shown, miR‐218‐5p deregulation is involved in GBM growth and migration potential. In addition to the direct influence that miR‐218 has on transcripts such as TN‐C or SDC‐2, as shown in this study, it can influence the ECM composition by targeting other molecules, for example the Wnt/β‐catenin pathway transcription factors *LEF1* or MMP‐9.^80^ There are data showing that miR‐218 suppresses cell invasion and spheroid formation,^81^ arrests GBM cells in G1 phase^31^ and can reduce the expression of cancer stem cell markers such as CD133, SOX2 and Nestin.^82^ The complex influence that miR‐218 has on GBM cells cannot be underestimated and studied only by evaluating direct targets of this miRNA; therefore in search of indirect targets of miR‐218 in glioblastoma, we performed an extended‐expression analysis and found 47 genes connected to focal adhesion and cell motility. After miR‐218 overexpression in glioblastoma cells, we observed a decrease in the expression levels of GBM oncogenes such as *PIK3CA*, *ROCK1*, *LAMC1* and *ICAM1*. The expression of these genes is increased in GBM compared with healthy tissues.^83^, ^84^, ^85^, ^86^ Enhanced miR‐218 levels also reduced the expression levels of the *CRK*, *RHOA* and *PTPN1* genes involved in GBM progression.^87^, ^88^, ^89^ Our results are supported by data in the literature indicating a decrease in the expression levels of *PIK3CA*,^90^ *RHOA* ^91^ and *STAT3* ^50^, ^92^ as a consequence of miR‐218 overexpression.

Due to the nature of our research, the changes in the expression levels of *CDC42*, *STAT3*, *EGF* and *CTTN* might be particularly important. Previous reports have indicated that *CDC42* is a critical determinant of the migratory and invasive phenotype of malignant gliomas.^93^, ^94^ The *STAT3* level is correlated with GBM malignancy, indicating its participation in increasing the migration potential of cancer cells.^95^ Additionally, regarding *EGF*, its impact on the migratory nature of GBM cells is known.^96^ *CTNN* and the Arp2/3 complex are known for regulating lamellipodia formation, and a decrease in CTNN expression can suppress GBM migration mechanisms.^97^, ^98^ Because we showed a decrease in the migration capacity of glioblastoma cells under treatment with miR‐218 in our studies, we could conclude then that these changes might be the result of the impact of miR‐218 on *CDC42*, *STAT3*, *EGF* and *CTTN*.

The observed increase in GBM cell adhesion may also be associated with a decrease in *ACTN1* expression. It has been shown that after downregulation of *ACTN1*, GBM cells show poor spread but increased focal adhesion.^99^ The changes in the cytoskeleton that we observed may be the result of a reduced *HGF* level, which has been demonstrated to affect the distribution of the actin cytoskeleton in glioblastoma cell lines.^100^ Both the cancer migration pathway and deregulation of the actin cytoskeleton can be related to downregulation of SH3PXD2A after miR‐218 overexpression. SH3PXD2A is a crucial element in the formation of actin‐based invadopodia—protrusions of the plasma membrane that are associated with mechanisms of invasiveness.^101^, ^102^

## 5 CONCLUSIONS

In this study, we showed that miRNA, as post‐transcriptional gene regulator, has a direct impact on the ECM composition and, as a consequence, the mechanobiological properties of glioma cells. We demonstrated that miR‐218 can be considered as a potent tumour suppressor that directly participates in post‐transcriptional regulation of the expression of the extracellular matrix proteins tenascin‐C and syndecan‐2. The most intriguing observations in this study are the impact of miR‐218 on the mechanical properties of the cells, that is migration and adhesion, followed by the direct changes of cell stiffness as measured with AFM technology. Additionally, our global gene expression analysis revealed changes in a number of genes directly or indirectly involved in cell motility and thus adhesion or cytoskeletal rearrangement. Taken together, our results showed the direct impact of miR‐218 on the qualitative ECM content, leading to changes in the rigidity of the ECM and GBM cells. These features impacted by miR‐218 overexpression collectively reduce the motility of cancer cells and increase their adhesiveness, thus most probably conferring a phenotype more closely related to that of normal cells. Collectively, our results indicate that miR‐218 is a potent tumour suppressor in glioma with a large impact on the ECM and biomechanical properties of the cells. Additionally, we believe that cell mechanical properties can constitute a broad drug target space, allowing possible corrective modulation of tumour cell behaviour.

## AUTHOR CONTRIBUTIONS

**Małgorzata Grabowska:** Conceptualization (equal); investigation (lead); visualization (equal); writing – original draft (equal); writing – review and editing (equal). **Konrad Kuczyński:** Investigation (lead); methodology (equal); software (equal); visualization (equal); writing – original draft (equal); writing – review and editing (equal). **Monika Piwecka:** Investigation (supporting); methodology (equal); writing – review and editing (equal). **Alicja Rabiasz:** Investigation (supporting). **Joanna Zemła:** Data curation (equal); investigation (supporting); methodology (equal); software (equal). **Paweł Głodowicz:** Investigation (supporting). **Dariusz Wawrzyniak:** Investigation (supporting). **Małgorzata Lekka:** Data curation (equal); methodology (equal); software (equal); writing – original draft (equal); writing – review and editing (supporting). **Katarzyna Rolle:** Funding acquisition (lead); project administration (lead); supervision (lead); writing – original draft (equal); writing – review and editing (equal).

## CONFLICT OF INTEREST

The authors confirm that there are no conflicts of interest.

## INSTITUTIONAL REVIEW BOARD STATEMENT

Tissue samples used in this study were collected based on the approval from the Karol Marcinkowski University of Medical Sciences in Poznan Ethical Committee (Consent nr. 46/13).

## Supporting information


Supplementary information 1

Supplementary information 2

Supplementary information 3

## Data Availability

Data available on request from the authors.
