# Supplementary material for: miR‐218 affects the ECM composition and cell biomechanical properties of glioblastoma cells
Source: J Cell Mol Med. 2022 Jun 15;26(14):3913–30. doi: 10.1111/jcmm.17428 (PMC9279592; doi:10.1111/jcmm.17428)
Supplement: Supplementary file 1 — Supplementary information 1 [file JCMM-26-3913-s002.docx]

| **gen** | **adnotacja RefSeq** | **ENCORI** | **miRDB** | **PicTar** | **TargetScan** |
| --- | --- | --- | --- | --- | --- |
| ATRN | NM_139321 | + | + | - | + |
| CDH2 | NM_001792 | + | + | + | - |
| CDH8 | NM_001796 | - | + | - | - |
| ELFN2 | NM_052906 | - | + | - | + |
| FLRT2 | NM_001346144 | + | - | + | + |
| HAPLN1 | NM_001884 | - | + | + | + |
| HTR7 | NM_000872 | - | + | + | - |
| NCAN | NM_004386 | - | + | - | + |
| PRG4 | NM_001127708 | - | + | - | - |
| RELN | NM_005045 | - | + | + | + |
| SDC2 | NM_002998 | **+** | **+** | **+** | **+** |
| SGCZ | NM_001322879 | - | + | + | + |
| TNC | NM_002160 | + | + | - | + |
