# Supplementary material for: miR‐218 affects the ECM composition and cell biomechanical properties of glioblastoma cells
Source: J Cell Mol Med. 2022 Jun 15;26(14):3913–30. doi: 10.1111/jcmm.17428 (PMC9279592; doi:10.1111/jcmm.17428)
Supplement: Supplementary file 3 — Supplementary information 3 [file JCMM-26-3913-s001.docx]

| gene | fold change | p-value | RefSeq record |
| --- | --- | --- | --- |
| ITGAV | 14,0419 | 0,049032 | NM_002210 |
| COL12A1 | 13,2538 | 0,043311 | NM_004370 |
| ITGA3 | 9,3718 | 0,03991 | NM_002204 |
| ACTN3 | 2,32668 | 0,037733 | NM_001104 |
| ITGB3 | 1,1824 | 0,045621 | NM_000212 |
| ICAM1 | -1,0527 | 0,049994 | NM_000201 |
| VIM | -1,16366 | 0,04722 | NM_003380 |
| FN1 | -1,1849 | 0,046053 | NM_002026 |
| RND3 | -1,2241 | 0,044132 | NM_005168 |
| TIMP3 | -1,26043 | 0,042594 | NM_000362 |
| LAMC1 | -1,34694 | 0,048150 | NM_002293 |
| WASF2 | -1,3645 | 0,045001 | NM_006990 |
| MET | -1,3771 | 0,013717 | NM_000245 |
| WIPF1 | -1,3835 | 0,020224 | NM_003387 |
| EGF | -1,3867 | 0,025987 | NM_001963 |
| FAP | -1,4093 | 0,016415 | NM_004460 |
| ITGA4 | -1,4093 | 0,025599 | NM_000885 |
| ROCK1 | -1,4126 | 0,006 | NM_005406 |
| FGF2 | -1,4126 | 0,014704 | NM_002006 |
| ARHGEF7 | -1,4224 | 0,00922 | NM_003899 |
| MYLK | -1,4257 | 0,017474 | NM_053025 |
| STAT3 | -1,4828 | 0,042723 | NM_003150 |
| MYH9 | -1,5245 | 0,021593 | NM_002473 |
| CFL1 | -1,5637 | 0,035133 | NM_005507 |
| ACTR3 | -1,5674 | 0,027278 | NM_005721 |
| MSN | -1,571 | 0,001185 | NM_002444 |
| ITGB1 | -1,6339 | 0,040562 | NM_002211 |
| TLN1 | -1,6876 | 0,000969 | NM_006289 |
| RASA1 | -1,7231 | 0,011208 | NM_002890 |
| HGF | -1,7634 | 0,031988 | NM_000601 |
| CDC42 | -1,7715 | 0,032539 | NM_001791 |
| RHO | -1,7839 | 0,044098 | NM_000539 |
| ACTR2 | -1,8553 | 0,004925 | NM_005722 |
| PTPN1 | -1,8682 | 0,000465 | NM_002827 |
| ACTN1 | -1,8682 | 0,01462 | NM_001102 |
| IGF1 | -1,9341 | 0,020683 | NM_000618 |
| TNC | -1,9852 | 0,031457 | NM_002160 |
| RHOA | -2,0162 | 0,048735 | NM_001664 |
| MYH10 | -2,035 | 0,021525 | NM_005964 |
| BAIAP2 | -2,1019 | 0,000482 | NM_006340 |
| PTK2B | -2,5111 | 0,018089 | NM_004103 |
| WASL | -2,5344 | 0,00463 | NM_003941 |
| CTTN | -2,7226 | 0,000898 | NM_005231 |
| CRK | -2,9383 | 0,00659 | NM_016823 |
| SH3PXD2A | -3,0209 | 0,000972 | NM_014631 |
| PTEN | -3.056 | 0,044654 | NM_000314 |
| PIK3CA | -3,6765 | 0,042649 | NM_006218 |
